# An abnormal clinical Allen's Test is not a contraindication for free radial forearm flap

**DOI:** 10.1002/ccr3.3093

**Published:** 2020-07-15

**Authors:** Travis J. Miller, Bauback Safa, Andrew J. Watt, Mang L. Chen, Walter C. Lin

**Affiliations:** ^1^ The Buncke Clinic San Francisco California USA; ^2^ Division of Plastic and Reconstructive Surgery Department of Surgery Stanford Hospitals and Clinics Palo Alto California USA; ^3^ G.U. Recon San Francisco California USA

**Keywords:** Allen's test, hand surgery, microsurgery, phalloplasty, radial forearm flap, transgender

## Abstract

An abnormal clinical Allen's test is not a definitive exclusion criterion for free radial forearm flap use. A surgical Allen's test may be useful to determine whether flap harvest is feasible in patients with an abnormal clinical Allen's test.

## Graphical abstract

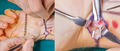

## 1 INTRODUCTION

The use of the clinical Allen's test is widely recommended prior to harvest of a radial forearm flap, and many practitioners advocate that an abnormal Allen's test is a contraindication to harvest of a radial forearm flap from that limb due to the risk of hand ischemia.^1^, ^2^, ^3^ In female‐to‐male gender affirming phalloplasty, the most commonly employed techniques are pedicled anterolateral thigh (ALT) flaps or free radial forearm flaps (FRF). ^4^ Published literature has established a superior complication profile in FRF compared with pedicled ALT flaps.^5^, ^6^ Thus, an abnormal Allen's test in a patient undergoing gender affirmation phalloplasty may unnecessarily preclude them from obtaining a FRF flap and subject them to a higher long‐term complication profile.

The goal of this study was to determine whether an abnormal Allen's test correlates with direct radial artery occlusion accessed surgically, and whether the Allen's test should continue to act as exclusion criterion for FRF.

## 2 MATERIALS AND METHODS

At a single institution, patients received consultation for gender affirming phalloplasty. Patients were offered pedicled ALT flaps or FRF flaps for phalloplasty. For FRF flaps, the donor site considered was the nondominant limb, and harvest from the dominant limb was not typically offered given potential donor site morbidity. All patients underwent a clinical Allen's test as part of their routine perioperative workup by the senior attending (who was a board‐certified hand and plastic surgeon) on the case. An Allen's test was considered abnormal whether perfused color did not return to the hand after release of the ulnar artery after 10 seconds.

For patients who strongly desired FRF flaps (which is common in transgender patients given implications on phalloplasty result and stigma for donor sites) despite an abnormal Allen's test, an alternative surgical plan was proposed compared with routine harvest of the radial forearm flap. Patients were counseled and consented for radial artery cutdown, and a “surgical Allen's test” would be performed to assess collateral flow to the hand. After extremity exsanguination and tourniquet inflation, the radial artery was exposed surgically and a vascular clamp was applied. After deflation of the tourniquet, hand and digit perfusion was assessed with return of color, turgor, capillary refill, and doppler ultrasound flow within the digits.

With normal collateral perfusion, FRF phalloplasty would proceed utilizing that limb. If the patient did not pass the surgical Allen's test, the flap would be aborted from that limb and an alternative flap would be utilized.

## 3 RESULTS

From 2016 to 2018, 123 FRF flaps were performed at our institution for phalloplasty reconstruction. Of these patients, four patients were found to have an abnormal Allen's test during preoperative consultation on the preferred donor limb. These patients were counseled as noted above and proceeded with a surgical Allen's test.

**Figure 1:**
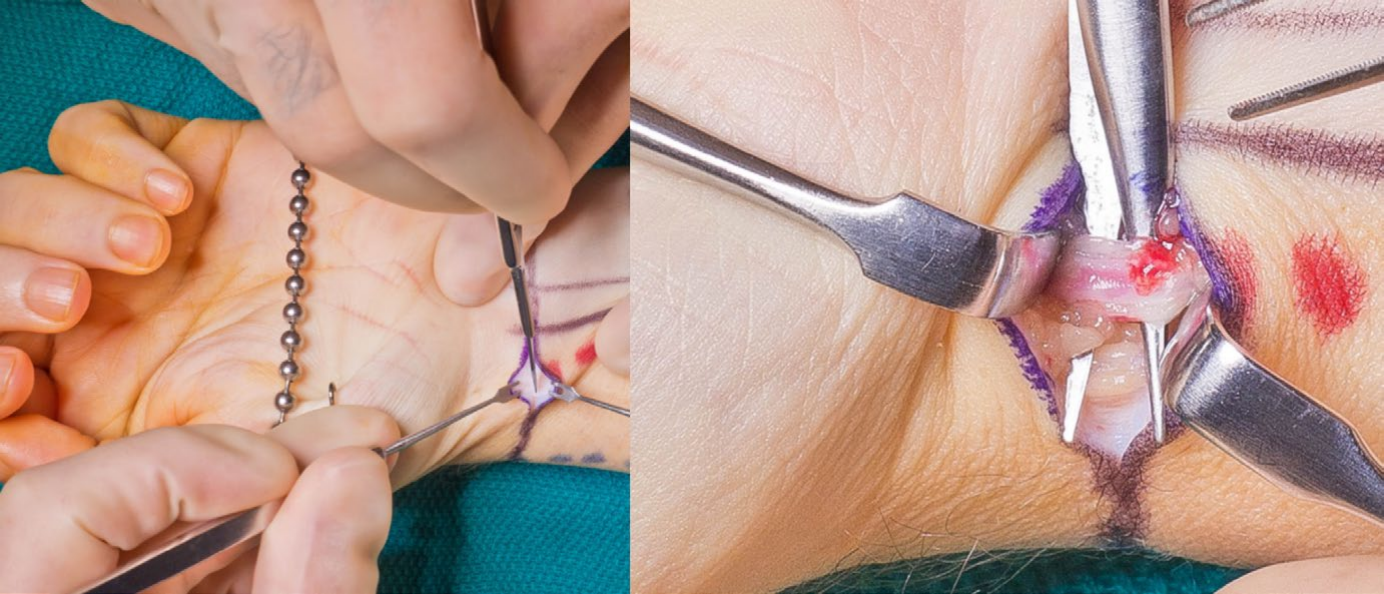
Radial artery cutdown. Left panel: Patients were marked for FRF phalloplasty preoperatively. Initial incision was performed at the radial artery to allow for direct occlusion and testing of hand perfusion prior to committing to raisings the flap. Right panel: Magnified view demonstrating isolation of the radial artery prior to placement of a vascular clamp

Three patients underwent radial artery cutdown on the left upper extremity, and one patient underwent cutdown on the right upper extremity. Representative photos of the cutdown technique can be seen in Figure 1. All patients underwent a surgical Allen's test and demonstrated adequate perfusion through the ulnar artery, and FRF flap harvest proceeded on the planned limb. Effects on Doppler ultrasound to the digits were not appreciable affected after clamping of the radial artery in these cases. The radial artery was not reconstructed for these patients. Surgical cutdown did not adversely affect flap elevation as the incision for the cutdown was incorporated into the design of the flap.

None of the patients who underwent free radial forearm phalloplasty after radial artery cutdown suffered ischemic consequences in the involved extremity. Furthermore, all patients recovered from phalloplasty without partial or total flap loss, and postoperative recovery remained uneventful.

**Figure 2:**
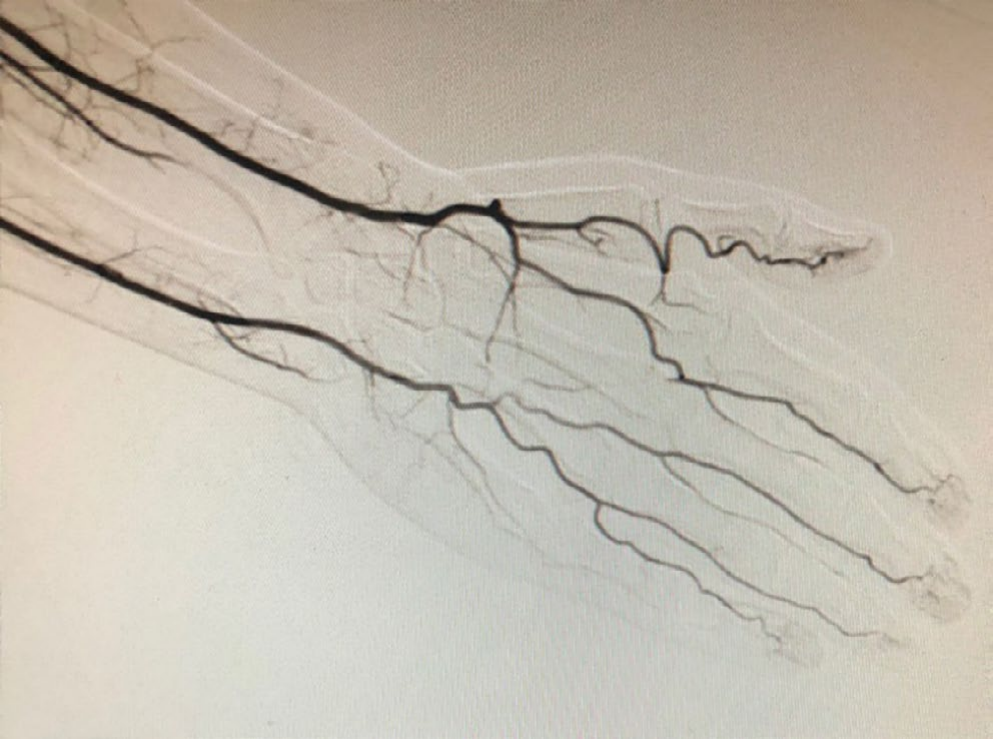
Left hand angiogram. This angiogram was obtained for one patient in the series. The angiogram demonstrates the lack of a superficial arch and the deep palmar arch is diminutive, suggesting that hand perfusion is co‐dominant between the radial and ulnar blood supplies. However, a radial forearm flap was successfully harvested for this patient after surgical Allen's test

**Figure 3:**
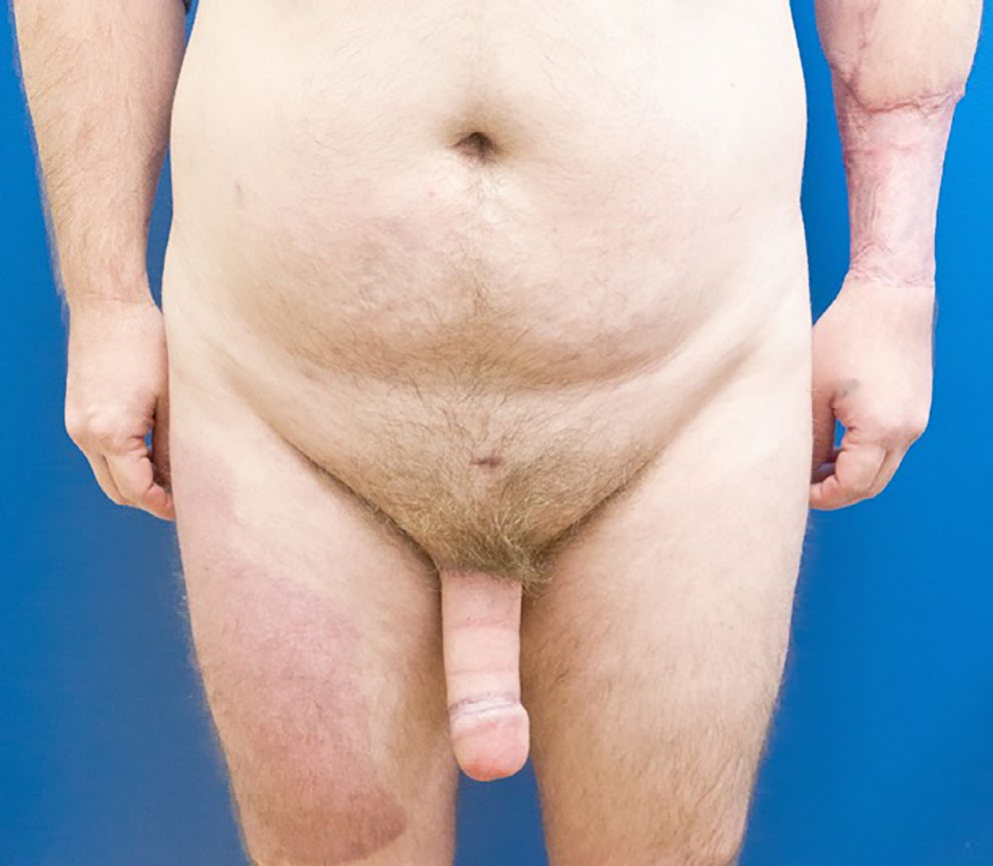
A 2‐y follow‐up after free radial forearm phalloplasty. The patient was satisfied with his result and noted no functional impairment with his left upper extremity

Notably, one patient received a left upper extremity angiogram prior to free radial forearm phalloplasty as part of their preoperative workup (Figure 2). The results of this angiogram suggested that blood flow to the hand was dependent on both the radial and ulnar arterial systems without an apparent superficial arch. However, the patient strongly desired FRF phalloplasty, and the intraoperative surgical Allen's test demonstrated adequate ulnar perfusion to the hand. The patient's subsequent recovery was uneventful, and he regained full function of the left hand without cold intolerance. Two‐year follow‐up demonstrated adequate healing of his phalloplasty and left upper extremity (Figure 3).

Follow‐up for all four patients exceeded one year. All patients noted full functional recovery with the operated limb, and no patient experienced cold intolerance of the operated extremity.

## 4 DISCUSSION

Our series suggests that the clinical Allen's test is not necessarily a reliable predictor of ulnar artery perfusion to the hand. While a normal Allen's test is often considered as necessary prior to radial forearm flap harvest,^7^ our series demonstrates this is not always the case. Similar studies have been performed in the head and neck literature; Wood et al^8^ had a similar approach in performing radial artery cutdowns as the “gold standard” for determining radial forearm flap candidacy. The mechanism of why a clinical Allen's test would be a false positive while the surgical Allen's test potentially has a higher specificity is not clear. However, surgical exposure of the radial artery with direct occlusion eliminates potential ambiguities of if a vessel is truly occluded or not as may be seen in a clinical Allen's test.

Limitations of this study include its small sample size for patients with an abnormal clinical Allen's test. Only four of the 123 patients (3.3%) in this study had an abnormal clinical Allen's test; while each of those patients had an acceptable result, it is reasonable to question if the study was larger if adverse effects of radial artery sacrifice may be seen. Further study is needed to determine the specificity and sensitivity of the surgical Allen's test described in this series. Ischemic consequences would be devastating and are unacceptable for an elective operation and would require emergent revascularization. However, profound digit ischemia would be less likely with demonstrated perfusion after radial artery cutdown. Less severe symptoms of impaired blood flow, such as cold intolerance, may be more common, but they were not seen in this set of patients (both those with a normal and abnormal clinical Allen's test).

Since the clinical Allen's test lacks reliable assessment of ulnar artery perfusion to the hand, less invasive adjunct testing would be desirable. Previous studies have shown that clinically performed ultrasound is not always reliable demonstrating dominant blood flow to the hand,^8^, ^9^ which is one reason this was not offered as an adjunct study for these patients prior to surgical Allen's test. Notably, standard angiography similarly did not serve as a reliable predictor of hand perfusion in the case described in this study, suggesting the typical angiogram does not account for dynamic changes in flow pattern that occur with radial artery occlusion. Arterial contrast from selective ulnar artery catheterization may misleadingly show poor radial‐sided perfusion unless the radial artery has been occluded. Modified angiograms with radial artery occlusion performed during the procedure may be beneficial, but further studies are needed to determine whether such imaging modalities would be of clinical utility.

## 5 CONCLUSIONS

The clinical Allen's test should not be an absolute contraindication to FRF phalloplasty. Intraoperative radial artery exposure for a surgical Allen's test may be a useful tool to assess for candidacy for FRF flap in select patients willing to undergo a radial artery cutdown. Alternative testing modalities with high specificity would be desirable to avoid such an invasive diagnostic test and should be investigated in more detail.

## CONFLICTS OF INTEREST

None declared.

## AUTHOR CONTRIBUTIONS

TJM: collected data, performed patient care, and wrote this manuscript. BS: performed patient care, collected data, and edited this manuscript. AJW: performed patient care and collected data. MLC: performed patient care and collected data. WCL: performed patient care, collected data, and edited this manuscript.

## ETHICAL APPROVAL

The authors of this manuscript attest that all methods conform to the guidelines set by the Declaration of Helsinki. All patients in this study provided informed consent to procedures performed.
